# The Prediction of Distribution of the Invasive *Fallopia* Taxa in Slovakia

**DOI:** 10.3390/plants11111484

**Published:** 2022-05-31

**Authors:** Petra Gašparovičová, Michal Ševčík, Stanislav David

**Affiliations:** 1 Institute of Landscape Ecology of Slovak Academy of Sciences, Akademická 2, 949 10 Nitra, Slovakia; stanislav.david@savba.sk; 2 Department of Ecology and Environmental Sciences, Faculty of Natural Sciences and Informatics, Constantine the Philosopher University in Nitra, Tr. A. Hlinku 1, 949 01 Nitra, Slovakia; msevcik@ukf.sk

**Keywords:** invasive plants, species distribution model, *Fallopia* taxa

## Abstract

Invasive species are now considered the second biggest threat for biodiversity and have adverse environmental, economic and social impacts. Understanding its spatial distribution and dynamics is crucial for the development of tools for large-scale mapping, monitoring and management. The aim of this study was to predict the distribution of invasive *Fallopia* taxa in Slovakia and to identify the most important predictors of spreading of these species. We designed models of species distribution for invasive species of *Fallopia*—*Fallopia japonica*—Japanese knotweed, *Fallopia sachalinensis*—Sakhalin knotweed and their hybrid *Fallopia* × *bohemica*—Czech knotweed. We designed 12 models—generalized linear model (GLM), generalized additive model (GAM), classification and regression trees (CART), boosted regression trees (BRT), multivariate adaptive regression spline (MARS), random forests (RF), support vector machine (SVM), artificial neural networks (ANN), maximum entropy (Maxent), penalized maximum likelihood GLM (GLMNET), domain, and radial basis function network (RBF). The accuracy of the models was evaluated using occurrence data for the presence and absence of species. The final simplified logistic regression model showed the three most important prediction variables lead by distances from roads and rails, then type of soil and distances from water bodies. The probability of invasive *Fallopia* species occurrence was evaluated using Pearson’s chi-squared test (χ${{}_{}^{2}}_{1}^{}$). It significantly decreases with increasing distance from transport lines (χ${{}_{}^{2}}_{1}^{}$ = 118.85, *p* < 0.001) and depends on soil type (χ${{}_{}^{2}}_{1}^{}$ = 49.56, *p* < 0.001) and the distance from the water, where increasing the distance decrease the probability (χ${{}_{}^{2}}_{1}^{}$ = 8.95, *p* = 0.003).

## 1. Introduction

Long-distance dispersal of species by human activities and biological invasions are a main component of global change of the world [1,2]. Invasive species have become a major challenge in protecting biodiversity in the new millennium [3] and one of the world’s most costly environmental problems [4]. For the past several decades, the invasive plant species have posed severe threats to the local biodiversity, ecosystem services, environmental quality [5,6,7,8] and human health [9,10,11]. Invasive plants are, simply by occupying a large amount of space in invaded habitats, expected to impose a significant impact on the native vegetation and their associated food webs [12]. Invasive species may have some qualities that are responsible for their invasive nature. Although it is to be expected that different characteristics will be important in different places, there are some general characteristics of invasive species: high population growth rate high dispersal, vegetative reproduction, the ability of a species to maintain itself until conditions are favorable. Other possible characteristics are a large native range, human commensalism, single-parent reproduction, high genetic variability, phenotypic plasticity and maybe many others [13,14].

In Europe *Fallopia* taxa show a strong preference for man-made habitats and localities along roads and watercourses. However, the hybrid *F*. × *bohemica* shows the highest proportion of localities outside human settlements [15]. Largescale invasion by this invasive species is therefore likely to seriously affect biodiversity and reduce the quality of riparian ecosystems [12]. The genus *Fallopia* includes three invasive taxa in Europe—*F. japonica*, *F. sachalinensis* and *F. × bohemica* [15]. Japanese knotweed (*Fallopia japonica* (Houttuyn) Ronse-Decraene; Polygonaceae was introduced as an ornamental plant in Europe in the 1840s and in North America in the 1870s; and since, it has spread throughout these continents [16,17]. Japanese knotweed may cross with giant knotweed (*F*. *sachalinensis* (F. Schmidt) Ronse-Decraene), forming a hybrid, Bohemian knotweed (*F*. × *bohemica* (Chrtek & Chrtkova) J. P. Bailey), which possesses higher invading capabilities than its parents and forms the majority of knotweed plants in many areas [18,19]. Representatives of the *Fallopia* taxa are able to grow in diverse soil types (e.g., sand, loams, peat, alluvial and colliery soils, clay, shingles), with various pH ranges and nutrient content [16]. They are even able to establish on soils with high concentrations of heavy metals [20,21] and Sulphur dioxide [16]. Richards et al. [22] reported clones of *Fallopia* species even on highly saline soils. However, the survival of juveniles seems to be impeded by low soil humidity and droughts [23], and likely by extreme temperatures [16,24]. The ability to regenerate from vegetative fragments and disperse via seeds, the ability to shade out competitors, and the ability to adapt rapidly through epigenetic change makes knotweed a formidable invader [19].

Species distribution models (SDMs) are numerical tools that combine observations of species occurrence or abundance with environmental variables. They are used to gain ecological and evolutionary insights and to predict species distributions across landscapes [25]. SDMs are among the most widely used in ecology and conservation science [25,26]. They have become the basic methodological framework for predicting the occurrence of non-native species and for assessing the impact of human activities on invasive species distribution [27] and becoming a tool for early detection and control of the spread of invasive species [28]. Predicting the probability of successful establishment of plant species by matching environmental variables has considerable potential for incorporation in early warning systems for the management of biological invasions [29]. Species distribution models are an increasingly important tool in conservation decision making. Predicting the spatial distribution of invasive plants, understanding the ecological requirements of those species and the different environmental drivers that influence their distribution can improve the management of species invasions [30,31].

Despite the invasion of *Fallopia* taxa is among the most intensively studied plant invasions globally [32], up until this point, there has been any research conducted on the modelling of spatial distribution of *Fallopia* species in Slovakia. The research that has been completed has not focused on species distribution modelling. Instead, it has focused on actual distribution or impact on ecosystems. Renco et al. [33] investigated the communities of soil nematodes in the forest habitats invaded and uninvaded by *Fallopia japonica* in Tatra National Park, Slovakia. Mereďa et al. [34] studied cytological and morphological variation of *Fallopia* taxa (Polygonaceae) in the Krivánska Malá Fatra Mountains. Cytological and morphological variation of *Fallopia* sect. Reynoutria taxa (Polygonaceae) in the Krivánska Malá Fatra Mountains. Changes in habitat conditions of invaded forest communities in Podunajská Nížina and the impact of non-native species on biodiversity was studied by Lukovičová et al. [35]. However, there are studies focused on distribution of *Fallopia* done in Europe. Jovanović et al. [36] made a case study from Southeastern Europe with the aim to predict in which habitats and along which corridors its future spread can be expected. Pěknicová et al. and Pěknicová and Berchová-Bímová [37,38] predicted the distribution of invasive species in Czech Republic where Fallopia was one of modelled species.

This study focuses on spatial prediction of distribution of three invasive *Fallopia* species in Slovakia. We performed several distribution models to model the potential distribution of these species and identified most important prediction variables, which can be drivers of species distributions.

## 2. Results

We performed several distribution models and select those with the best prediction power based on AUC criteria (Table 1). The general problem with species distribution models is that there will always be variation between variability in results between different methods, without any unambiguous indicators of which model is the right one. A possible solution to account for this inter-model variability is to fit ensembles of forecasts by simulating across more than one method.

**Figure 1:**
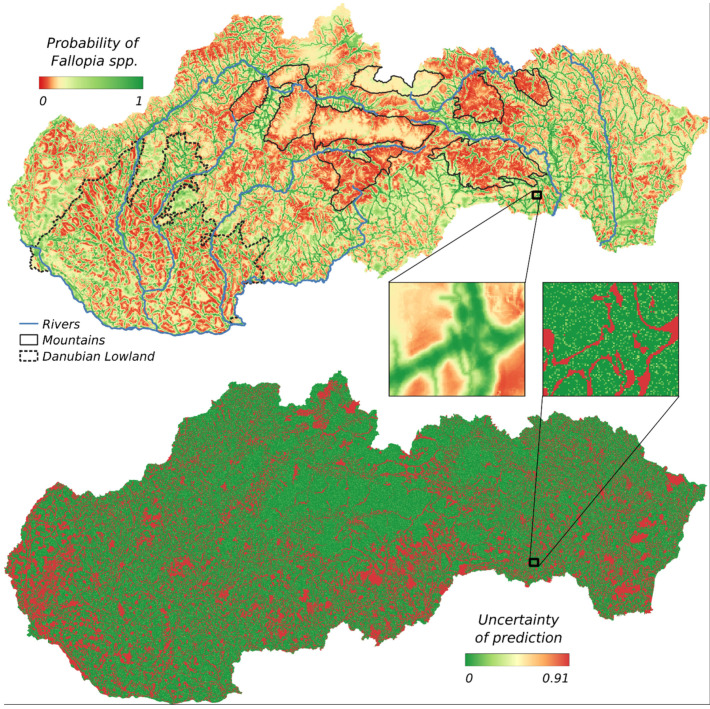
The ensemble prediction model of *Fallopia* species., with selected components and the uncertainty of a prediction. Selected components represent geomorphological units with relative homogenetic *Fallopia* species predictions.

Models from Table 1 served as the input for the final ensembled prediction model (Figure 1). The model shows the probability of invasive *Fallopia* species distribution in the area of Slovakia. The highest probability of occurrence is along the roads and rivers, as they represent the corridors of invasive species spreading. The Southern Slovak Basin and Košice Basin (situated in the east of Slovakia) are parts with the highest chance of occurrence of *Fallopia* taxa. The map shows the link between the road density and the occurrence of this invasive species in the area of southwestern Slovakia located in the Danubian Lowlands. Areas without a dense road network are least likely to occur *Fallopia*. Effect of the river network is clearly shown on the map. Rivers also present corridors of spreading *Fallopia* species in Slovakia and the river network forms continuous area with the high probability of spreading the species. This probability is highest on the river Váh and Hron. The lower probability of presence is also related to mountain areas. This can be caused not just by the higher elevation, but also by the presence of the protected areas and national parks, where invasive species are removed.

In addition to the prediction map, Figure 1 also shows the uncertainty of the prediction of the ensembled model. The greatest uncertainty can be seen in areas with the mean values of probability, outside the limit values (0 or 1). It was in these places that there was the most often discrepancy/indecision of individual models. The overall uncertainty of prediction (proportion of cells with an uncertainty of more than 50%) was 34%.

**Figure 2:**
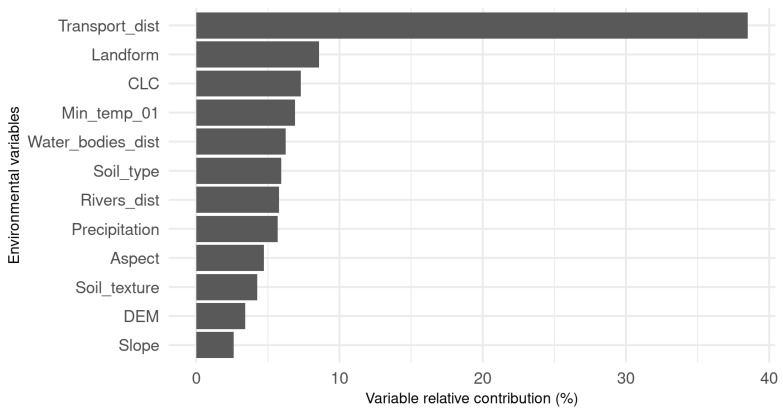
Ensemble model environment variable importance evaluated with Pearson.

Secondary output of distribution modeling (Figure 2) is also the evaluation of variable importance (computes as the difference between a full model and one with each variable successively omitted). This evaluation pointed out more important variables, which has a higher impact on prediction. Because of a good prediction result of GLM, we produced logistic regression, for better reproducibility and simpler further prediction of *Fallopia* spp. This model helps to investigate the probability of the occurrence of a dichotomous dependent variable by fitting the log odds and independent variables to a linear model, which are easy to interpret.

The final simplified logistic regression model showed the three most important prediction variables lead by distances from transport lines (roads and rails), then the type of soil (fluvisols, haplic luvisols, leptosols, mollic fluvisols and mollic gleysols, planosols and stagnosols) and the distances from the water bodies (Table 2).

The tested predictive power of this model achieved 0.91 AUC what is comparable with the result from the ensembled model.

**Figure 3:**
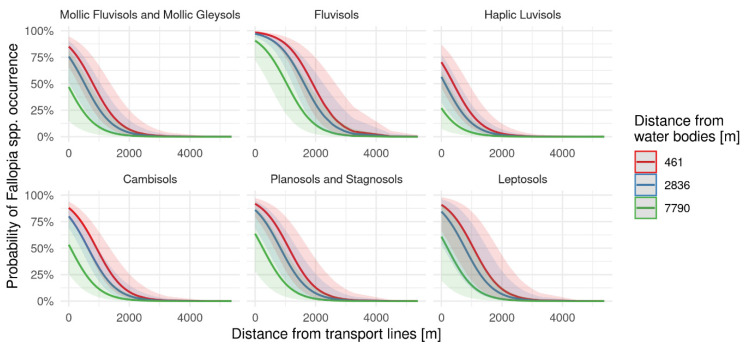
Modelled probability of *Fallopia* spp. based on distance from transport lines, water bodies and different soil types. Water bodies distances represent 10% (red), 50% (blue) and 90% (green) percentile.

The probability of *Fallopia* spp. occurrence significantly decreases with increasing distance from the transport lines (χ${{}_{}^{2}}_{1}^{}$ = 118.85, *p* < 0.001; Figure 3). Probability also depends on soil type (χ${{}_{}^{2}}_{5}^{}$ = 49.56, *p* < 0.001), being the highest is on Fluvisols. Less but still significant is the distance from the water, where increasing the distance decrease the probability (χ${{}_{}^{2}}_{1}^{}$ = 8.95, *p* = 0.003).

The final simplified logistic regression model showed natural disturbance (rivers) or anthropogenic disturbance (roads and rails) plays a role in explaining the presence of *Fallopia* spp. in Slovakia. The most important biophysical factor from used environmental variables are types of soil.

## 3. Discussion

The spread of invasive species depends on several ecological factors, the most important of which are environmental requirements, nutrient saturation, composition of invaded community, distance from roads and rivers, and effect of human activities [39]. Our results suggest that distance from transport lines, soil type and distance from water bodies are the most important factors for predicting the distribution of *Fallopia* taxa.

In this study, we designed distribution models of three invasive *Fallopia* species for the area of Slovakia to predict their spread. By our field mapping, we obtained current presence and absence data on selected transects. Habitat suitability predictions from SDMs are typically based on species occurrence data and are essentially occurrence probability or habitat suitability estimates. However, SDMs often do not involve true absence information, as confirmed absences are typically unavailable in most survey and monitoring databases. Given this difficulty to obtain absence information, several presence-only SDM approaches have been developed. The use of pseudoabsences involves many assumptions and needs careful model planning [40,41], and it is not surprising that studies generally suggest using absence data whenever they are available [28,42]. Another reason for using true absence data is to avoid uncertainty of the model. A major criticism and source of uncertainty in species distribution models is the lack of true absence information for accurate species distribution predictions [43,44]. The overall uncertainty of prediction (proportion of cells with an uncertainty of more than 50%) was 34%.

### 3.1. Distance from Transport Lines

Distance from roads and railways was the most important factor for *Fallopia* taxa spreading. Their presence along roads and railway is very frequent in Slovakia. Roads are especially well-documented sites for exotic plant invasion [45,46] and represent obvious dispersal corridors in a landscape [47]. They serve multiple functions that enhance exotic species invasion in this landscape: they act as corridors for dispersal, provide suitable habitat, and contain reservoirs of propagules for future episodes of invasion. [47]. Close associations between invasive *Fallopia* species and human disturbance along rail or road infrastructures have been reported [23,48] The occurrence of knotweeds is closely related to human-derived pressures [49]. Otherwise, roadside soils often contain high concentrations of heavy metals, released from fuel burning, wear out of tires, leakage of oils, and corrosion of car metal parts [50] and this pollution by metals may promote the clonal growth of *Fallopia* taxa [21]. This can lead to building their own environmental niche and thus favor their own expansion [51,52,53].

### 3.2. Soil Type

In our model, probability of *Fallopia* taxa occurrence also depends on soil type, being the highest on Fluvisols. Many studies do not consider soil type as an important predicting variable because *Fallopia* species have the ability to live in variety of soil types and varying levels between a pH of 3.0–8.5 [36]. Other studies, which included soil type variables in modelling process [21,37], suggest that soil type is one of the most important factors for predicting distribution of this species. One of the key mechanisms of plant invasiveness of *Fallopia* taxa is allelopathy—chemically mediated interference between plants, whereby secondary compounds produced by *Fallopia* species directly or indirectly (through affecting soil biota) suppress the growth and fitness of other species [54]. *Fallopia* is more allelopathic when resources of nutrients are abundant, and this may contribute to their superiority in nutrient-rich soils [55]. Its regeneration from fragments is affected by edaphic properties, with lower regeneration rates in poor soils [48,56]. Possibly because *Fallopia* species are usually dispersed on much richer soils [57]. Another explanation of the soil type variable importance could be using fine scale environmental layer and validated presence and absence data. Fine-scale data helps identifying conditions with the highest probability of invasion [58] and grain size smaller than 1 km should be preferred in SDM studies. However, models using finer grain size data should be trained and validated with carefully validated occurrence records [59].

### 3.3. Distance from Water Bodies

Less but still significant variable was the distance from the water bodies (rivers, streams), where increasing the distance decreased the probability of spreading *Fallopia* taxa. Riparian zones along rivers may serve as corridors for dispersal of exotic species [60,61] and may facilitate invasion both by providing corridors in a landscape and by creating disturbance [47]. High levels of invasion are found especially in lowland sandy areas and river corridors [62] as they may contribute to the dispersal of alien propagules [63]. *Fallopia* species can regenerate from stem fragments, and this regeneration is increased when they spent some time in water, what is potentially highlighting the role of hydrochory in the evolutionary history of this species [48,64,65]. They often form dense stands along rivers and have negative impacts on biodiversity and ecosystem functions and also threaten the stability of river banks [66]. *Fallopia* rhizomes both displace roots and the structure they provide to soil, also amplify bank-erosion forces [67]. Due to their life form, vitality and their enormous ability to regenerate themselves, they are extremely hard to fight [66].

A practical application of species distribution models might involve identifying environmental drivers of species distribution and abundance and predicting locations of high invasion risk [68] Predicting the probability of successful establishment of plant species has considerable potential for incorporation in the management of biological invasions [29]. SDMs have been extensively used to predict the potential geographical ranges of invasive species and some of those were used to predict distribution of *Fallopia* species. Jovanović et al. [36] predicted the future range of invasive *Fallopia* species in Southeast Europe. The results of this study predict the most suitable range for *F. japonica* and *F. sachalinensis* (in the north of the region studied) and for *F.* × *bohemica* (central Southeast Europe). This study predicts that *Fallopia* species could expand their range in riparian habitats up to 30–40%. Pěknicová et al. [37] constructed local SDMs for invasive alien plant species in the Kokořínsko Protected Landscape Area in Czech Republic. Bourchier at al. [69] conclude existing knotweed sites occupy just over half of the suitable habitat in British Columbia, indicating there are still significant areas to be invaded.

In summary, we believe that species distribution models can provide useful tool for invasive species management, and this study and the distribution map can provide insight for to guide decisions regarding prevent and control the spread of invasive *Fallopia* species.

## 4. Materials and Methods

### 4.1. Study Area

This study was conducted over entire Slovakia for following reasons: (i) *Fallopia* species create populations throughout Slovakia, (ii) the availability of all literature-recommended GIS layers needed for the modelling, and (iii) with regard to Slovak legislation, mandating the removal of these species from both public and private land, the creation of a prediction model provides useful information for species management at the regional and local level.

Slovakia is land-locked country in Central Europe and covers an area of 49,034 km². This country belongs to regions with variable environmental, geological, geomorphological and climate conditions. Most territory of Slovakia, especially the northern and central mountain areas, are in the Carpathians biogeographical region (moderately warm and cool regions with daily maximum air temperature ≥ 16 °C and <16 °C, respectively). The remaining areas lie in the Pannonian lowland plain (warm region with more than 50 summer days annually in average and daily maximum air temperature ≥ 25 °C). Relatively base-rich bedrocks, mainly limestone, various limnic and marine sediments as well as volcanic bedrocks such as andesite, form the majority of the Slovak territory. Acidic bedrocks are less frequent, occurring in high mountains (e.g., Tatry Mountains, Slovenské rudohorie Mountains) and in some of the flysc series of the Western Carpathians [70]. Geographical location, vertical differentiation, diverse geological substrate and rugged relief provide suitable conditions for the spread of many species.

### 4.2. Fallopia Occurrence Data

The base of the occurrence data was based on the *Fallopia* species database, composed from a long-term mapping of the state nature conservancy of Slovakia, literary sources and phytological research [71]. Due to the fact that the occurrence data in this database do not come from the same time period, they were not obtained by the same mapping method and contain only information about the presence of the species, we decided to verify the accuracy of the data and add the absence of the species with own terrain mapping.

**Figure 4:**
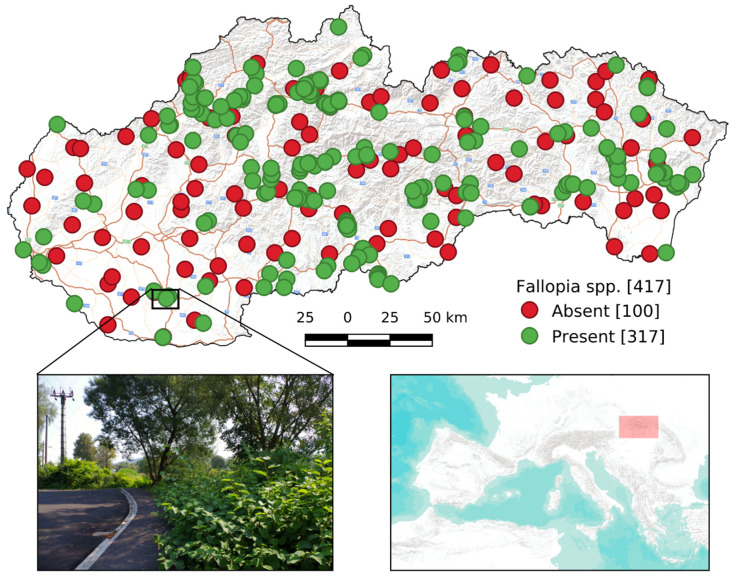
*Fallopia* taxa occurance data used in analysis.

To confirm the current presence of *Fallopia* species were randomly selected 100 georeferenced records from the database from a long-term mapping. These points from the database represents the central points of the presence transects, which confirm the accuracy of the database. To confirm the absence of the species, we designed another 100 transects. Both the presence and absence transect were 1 km long and 60 m wide. The occurrence of *Fallopia* species was mapped in the field research performed in June–October 2019. A total of 417 records were used for modeling, 317 of those represent the presence of the species and 100 represent the absence of the species (Figure 4). In all subsequent analysis, the occurrence/absence data were represented as center points of transects.

### 4.3. Environmental Data

The selection of environmental variables was based on a literature study of the properties and environmental requirements of invasive species of the genus *Fallopia* and of existing distribution models [16,36,37,38,72]. We collated 19 environmental predictors from multiple sources that provide the characteristic landscape conditions environmental requirements of *Fallopia* species.

All input layers were reprojected to the local coordinate system (EPSG:5514). Vector layers were converted to rasters and resampled and aligned to 50 × 50 m spatial resolution. To better capture, the influence of layers elements that did not directly overlap with occurrence data, were derived proximity layers indicating the distance from the center of each pixel representing a given element to the center of all surrounding pixels. To improve the fit of certain algorithms (such as artificial neural networks) were continuous variables normalized between 0 and 1 by divide by maximum value of each variable. All the raster layers were then collected into a multilayer raster stack. The environmental variables were processed in QGIS (QGIS.org, 2021).

Firstly, all available and literature-recommended environmental variables (19 layers) were tested for multicollinearity with variance inflation factor (VIF). Highly correlated variables with VIF higher than 5 were excluded from the analysis through a stepwise procedure. The remaining 12 environmental variables can be found in Table 3 (those excluded were bioclimatic factors such as annual mean temperature, annual temperature range, the maximum temperature of the warmest month and the minimum temperature of the coldest month).

### 4.4. Ensemble Distribution Model Development and Evaluation

Occurrence data were randomly split to the train dataset (70% of data) used for fitting models and test dataset for evaluation (30%). At first, the 12 most-used, standalone machine learning and statistical methods for species distribution modelling were fitted: generalized linear model (GLM), generalized additive model (GAM), classification and regression trees (CART), boosted regression trees (BRT), multivariate adaptive regression spline (MARS), random forests (RF), support vector machine (SVM), artificial neural networks (ANN), maximum entropy (Maxent), penalized maximum likelihood GLM (GLMNET), domain, and the radial basis function network (RBF). We applied the default parameters for all models, in line with typical usage of sdm package [69]. Each model was evaluated against training data, using 10 runs of 5-folds cross-validation replication methods (XXX models in total). Models which meet >0.9 AUC (area under the curve) criteria on the test dataset determinate were subsequently used to build the ensemble model (Table 1). The final model assembling was achieved in two steps: (1) by averaging the predictions of partial models (for every run and every replication) for each algorithm; (2) by calculating the weighted mean based on the AUC statistics for every algorithm from the first step. To determine the importance of predictor variables in explaining the species distribution were used a randomization procedure that measures the correlation between the predicted values and predictions where the variable under investigation is randomly permutated. If the contribution of a variable to the model is high, then it is expected that the prediction is more affected by a permutation, and therefore, the correlation is lower. Therefore, ‘1–correlation’ can be considered as a measure of variable importance [73,74]. Alongside the ensembled model, the uncertainty map among partial model predictions were also calculated. It ranges between 0 and 1, where 0 means all the models predicted the same value (either presence or absence), and 1 refers to maximum uncertainty = inconsistency among different models. Distribution models were produced an R environment [75].

### 4.5. Logistic Regression

To simplify the final predictions, restricted AIC-based stepwise logistic regression with Bernoulli error distribution and logit link function was used. For model improvement, were filtered out levels of categorical variables with less than 10 records. To test non-linear responses, the continuous variables used second-degree polynomials. The deviance table was tested by chi-square statistics. To compare the model’s predictive power as objectively as possible, AUC statistics from 100 randomly sampled models (70% train, 30% test) were averaged. All statistical tests were performed in an R environment [75].

## 5. Conclusions

We used 12 models to design the final simplified logistic regression model, which showed the 3 most important prediction variables lead by distances from roads and rails, then by type of soil, with the highest being Fluvisols, and distances from water bodies. The probability of invasive *Fallopia* species occurrence significantly decreases with increasing distance from transport lines and depends on soil type and the distance from the water, where increasing the distance decreases the probability. Roads and rivers provide not just a suitable habitat but present corridors of spreading this invasive species in Slovakia. The probability of *Fallopia* taxa occurrence along these structures is very high—highest on the river Váh and Hron. Our distribution model also showed areas such basins as the areas with the highest probability of occurrence of invasive *Fallopia* species. The highest probability of spreading this species was in the Southern Slovak Basin and the Košice Basin. The probability of distribution is lowest in the mountain areas of Slovakia, what can be caused not just by the higher elevation, but also by the presence of the protected areas and national parks, where invasive species are removed.

In summary, we believe that species distribution models can provide useful tool for invasive species management, and this study and the distribution map can provide insight for to guide decisions regarding prevent and control the spread of invasive *Fallopia* species. Given that environmental monitoring of invasive species and their next removal is very costly and sometimes simply impossible in the case of a large area, the model of *Fallopia* species distribution could provide an operational tool for such decisions

## Figures and Tables

**Table 1:** Accuracy evaluation statistics of models used for ensemble model.

Method	AUC	COR	TSS	Deviance
glm	0.90	0.63	0.7	0.75
rf	0.94	0.76	0.82	0.53
maxent	0.94	0.73	0.79	0.92
glmnet	0.92	0.55	0.77	2.78
brt	0.94	0.74	0.8	0.66
svm	0.92	0.69	0.76	0.65
mars	0.93	0.75	0.8	0.62
rbf	0.90	0.62	0.71	0.76
gam	0.93	0.73	0.79	0.87
ranger	0.94	0.75	0.81	0.52

**Table 2:** Coefficient table of logistic regression model.

Term	Estimate	Std. Error	Statistic (z Value)	*p*-Value
(Intercept)	2.24	0.417	5.39	7.21 × 10^−8^ ***
Distance from transport lines	−0.00222	0.000339	−6.56	5.29 × 10^−11^ ***
Soil type: Fluvisols	2.17	0.451	4.82	1.40 × 10^−6^ ***
Soil type: Haplic Luvisols	−1.25	0.570	−2.20	2.81 × 10^−2^ *
Soil type: Leptosols	0.203	0.848	0.239	8.11 × 10^−1^
Soil type: Mollic Fluvisols and Mollic Gleysols	−0.363	0.611	−0.594	5.52 × 10^−1^
Soil type: Planosols and Stagnosols	0.681	0.585	1.17	2.44 × 10^−1^
Distance from water bodies	−0.000250	0.0000875	−2.85	4.34 × 10^−3^ **

Statistically significant differences at: * *p* < 0.05; ** *p* < 0.01 and *** *p* < 0.001.

**Table 3:** List of used environment variables with variable inflation factor (VIF).

ID	Layer	Description	Type	VIF	Source
1.	Transport_dist	Euclidean proximity map of roads and rails (range: 10,573 m; mean: 1159 ± 1190 m)	Continuous	1.64	Institute of Landscape Ecology of SAS
2.	Aspect	Categorized aspect directions	Categorical (*n* = 8)	1.01	Derived from DEM
3.	CLC	CORINE Land Cover 2018 (hierarchical 3-level CLC nomenclature)	Categorical (*n* = 31)	1.44	EEA (2018)
4.	Landform	Type of slope landform	Categorical (*n* = 36)	1.63	Institute of Landscape Ecology of SAS
5.	Soil_texture	Soil texture	Categorical (*n* = 12)	1.08	Institute of Landscape Ecology of SAS
6.	Soil_type	Soil type	Categorical (*n* = 22)	1.42	Institute of Landscape Ecology of SAS
7.	Rivers_dist	Euclidean proximity to rivers (range: 6937 m; mean: 348 ± 390 m)	Continuous	1.25	Institute of Landscape Ecology of SAS
8.	DEM	Digital elevation model (range: 2521 asl; mean: 454 ± 313 m asl)	Continuous	4.08	EEA (2018)
9.	Slope	Surface slope (range: 76°; mean: 9 ± 8° m)	Continuous	1.62	Derived from DEM
10.	Water_bodies_dist	Euclidean proximity map of waterbodies (range: 17,430 m; mean: 3228 ± 2219 m)	Continuous	1.18	Institute of Landscape Ecology of SAS
11.	Min_temp_01	Minimum temperature in January (range: 7 °C; mean: −8±1 °C)	Continuous	2.42	Fick and Hijmans, 2017 (WorldClim)
12.	Precipitation	Precipitation (range: 1184 mm; mean: 734 ± 169 mm)	Continuous	2.57	Fick and Hijmans, 2017 (WorldClim)

## Data Availability

Not applicable.
